# Metabolic syndrome correlates intracoronary stenosis detected by multislice computed tomography in male subjects with sleep-disordered breathing

**DOI:** 10.1186/1758-5996-4-6

**Published:** 2012-03-01

**Authors:** Tomoko Nakanishi-Minami, Ken Kishida, Yasuhiko Nakagawa, Munetaka Nishio, Chisa Nakagawa, Yoshiharu Nishida, Koji Yanagi, Ryoko Yoshida, Tohru Funahashi, Iichiro Shimomura

**Affiliations:** 1 Department of Metabolic Medicine, Graduate School of Medicine, Osaka University, Suita, Osaka 565-0871, Japan; 2 Department of Metabolism and Atherosclerosis, Graduate School of Medicine, Osaka University, Suita, Osaka 565-0871, Japan; 3 Department of Cardiology, Kenporen Osaka Central Hospital, Kita-ku, Osaka 530-0001, Japan; 4 Department of Internal Medicine, Kenporen Osaka Central Hospital, Kita-ku, Osaka 530-0001, Japan; 5 Yoshida Suimin-kokyu Clinic, Nishi-ku, Osaka 550-0011, Japan; 6 Department of Metabolism and Atherosclerosis, Graduate School of Medicine, Osaka University, 2-2 B-5, Yamada-oka, Suita, Osaka 565-0871, Japan

**Keywords:** Coronary Stenosis, Sleep apnea-hypopnea, Visceral fat, Adiponectin

## Abstract

**Background:**

Sleep-disordered breathing (SDB), especially obstructive sleep apnea (OSA), has frequent complications include hypertension, dyslipidemia and insulin resistance based on abdominal obesity or excess visceral fat (called Syndrome Z). OSA is a potential risk factor for cardiovascular diseases. The clinical characteristics of Japanese OSA subjects with OSA remain unclear. The present study investigated prevalence and predictive factors of intracoronary stenosis detected by multislice computed tomography (MSCT) in Japanese male subjects with SDB/OSA.

**Findings:**

The study (O-VFStudy) subjects were 39 Japanese men with SDB/OSA who underwent all-night cardiorespiratory monitoring with fully attended polysomnography, and moreover both fat computed tomography (CT) scan and 64-row MSCT coronary angiography. The prevalence of coronary stenosis in this selected population with SDB/OSA was 15%. Logistic regression analysis showed a significant relationship between age-adjusted CAD and metabolic syndrome (*p *< 0.05), but not serum adiponectin levels and nocturnal fall in adiponectin. Subjects with the metabolic syndrome had significantly higher prevalence of CAD (31.3 versus 4.3%, *p *= 0.033), and lower levels of serum adiponectin (4.5 ± 0.6 versus 6.4 ± 0.6 μg/mL, *p *= 0.014), compared with groups without the metabolic syndrome.

**Conclusions:**

The present study describes that the prevalence of greater than 50% intracoronary stenotic lesions detected by MSCT was 15% and the metabolic syndrome was correlated with intracoronary stenosis detected by MSCT in Japanese SDB/OSA subjects.

**Trial Registration:**

UMIN 000002997

https://upload.umin.ac.jp/cgi-open-bin/ctr/ctr.cgi?function=brows&action=brows&type=summary&recptno=R000003633&language=E.

## Background

Sleep-disordered breathing (SDB), especially obstructive sleep apnea (OSA), is a potential risk factor for cardiovascular diseases, including coronary artery disease (CAD) and stroke [1-3]. OSA is common in middle-age men [4], and frequent complications include hypertension, dyslipidemia and insulin resistance based on abdominal obesity or excess visceral fat [5-8]. Visceral fat is a huge endocrine organ producing a variety of bioactive substances, conceptualized as 'adipocytokines' [9]. Our group discovered adiponectin as an adipocytokine in the human adipose cDNA library [10], which has anti-atherosclerotic property [11]. We have recently demonstrated that, 1) production of adiponectin is dysregulated in intra-abdominal obese patients with severe OSA [12,13], and 2) night-time onset of acute coronary syndrome is associated with excess visceral fat and SDB [14], which is called "Syndrome Z" [15]. However, there is little information on clinical features of Japanese OSA subjects with CAD.

The present study investigated prevalence and predictive factors of intracoronary stenosis detected by multislice computed tomography (MSCT) in Japanese male subjects with OSA.

## Methods and procedures

### Participants

Subjects were recruited from 50 consecutive Japanese male subjects with a history of snoring and/or witnessed apneas, who underwent all-night cardiorespiratory monitoring in the Yoshida Suimin-kokyu Clinic (Osaka, Japan) and had no history of CAD, which was defined as documentation of medication and history of myocardial infarction, angina, and/or previous coronary intervention. The study (O-VFStudy) subjects comprised 39 Japanese, who moreover underwent both fat computed tomography (CT) scan and 64-row MSCT coronary angiography (GE-LightSpeed VCT) in Kenporen Osaka Central Hospital (Osaka, Japan), excluding subjects with contrast-allergic disease and/or renal dysfunction (serum creatinine > 1.5 mg/dL) (n = 6), and apnea hypopnea index (AHI) < 5 events/hour (n = 5). The study was approved by the human ethics committee of each participating hospital and informed consent was obtained from each participant.

### Cardiorespiratory monitoring

All participants underwent overnight cardiorespiratory monitoring with fully attended polysomnography (Alice^®^4 Diagnostics Sleep System, Philips Respironics). The recorded signals were analyzed for the number of apneas and hypopneas. Apnea represented a decrease in the amplitude of airflow signal or thoracoabdominal motion decreased to < 10% of the baseline for at least 10 seconds. Hypopnea was defined as a decrease in airflow or respiratory effort to < 70% of baseline for at least 10 seconds associated with > 4% desaturation but did not meet the criteria for an apnea. The AHI was defined as the total number of apneas/hypopneas per hour of sleep time [16,17]. An AHI ≥ 5 established the diagnosis of SDB. All recordings were scored manually by an experienced polysomnographic technologist. The Epworth Sleepiness Scale (ESS) was used to report subjective day-time sleepiness [18]. Excessive daytime sleepiness was defined as an ESS score greater than 10.

### CT images

Total fat area, visceral fat area and subcutaneous fat area were computed and measured automatically using commercial software on CT scans taken at the umbilical level in a supine position (120 kV, 400 mAsec, section thickness of 5-10 mm, field of view of 400 mm, window width of 800-1000 Hounsfield units). All MSCT angiograms were scanned by trained technicians, according to a standard protocol. All MSCT angiograms were evaluated by at least two experienced examiners. The presence of stenosis with coronary plaque was evaluated visually on axial and cross-sectional images and curved multiplanar reconstructions. Intracoronary lesion was identified as atherosclerotic stenosis and/or highly calcification lesion of at least one segment of a major coronary artery confirmed by MSCT coronary angiograms, which is greater than 50% stenosis as a candidate for revascularization [19,20]. Patients with intracoronary lesion were considered to have CAD.

### Anthropometry and laboratory measurements

Height (cm), weight (kg) (and body mass index in kg/m^2^), neck circumference at the level of the cricothyroid cartilage, waist circumference at the umbilical level and hip circumference horizontally at the level of the greater trochanter of the femur, were measured in the standing position. Systolic and diastolic blood pressures were measured with a standard mercury sphygmomanometer on the right or left arm after the subjects had rested in a sitting position for at least 10 minutes. Smoking habit was documented on the routine baseline questionnaire. Metabolic syndrome was defined according to the Japanese criteria [21].

Venous blood samples were collected in the morning after overnight fast for measurements of glucose, lipids, and immunoreactive insulin. In each sleep study that included high-sensitivity C-reactive protein (hsCRP) and adiponectin monitoring, venous blood samples were obtained after wake-up while the subject was in the supine position. For the purpose of the present study, serum samples that were obtained at baseline from each study participant and stored at -20°C, were thawed and mesured for serum hsCRP (N-Latex CRP II, Dade Behring Inc, Marburg, Germany, intra-coefficients of variation (CV); 2.0%, inter-CV 2.3%) and adiponectin concentrations (Otsuka Pharmaceutical Co., Tokushima, Japan, intra-CV; 3.0%, inter-CV 5.1%) [12-14,22]. Serum concentration of thiobarbituric acid-reacting substance (TBARS), an important biomarker of systemic oxidative stress reflecting serum lipid peroxidation products, was determined by the method of Yagi (Japan Institute for the Control of Aging, Nikken SEIL Co., Shizuoka, Japan), as reported previously by our group [14,22].

### Statistical analysis

The correlations between CAD and clinical features were first analyzed by simple logistic regression analysis. In all cases, *p *values < 0.05 were considered statistically significant. All analyses were performed with the JMP Statistical Discovery Software 9.0 (SAS Institute, Cary, NC).

## Results

### Characteristics of male subjects with SDB/OSA

Of consecutive 50 subjects with suspected sleep apnea, data from 39 subjects (mean AHI 39 ± 4 events/h) were studied. The baseline characteristics of 39 subjects are listed in Table 1. The percentage of patients with excessive daytime sleepiness (ESS > 10) was 64% (n = 25/39). Only 15% (n = 6/39) of the subjects showed greater than 50% intracoronary stenotic lesions detected by MSCT. Furthermore, the affected coronary artery was the left anterior descending artery (5 lesions), left circumflex artery (1 lesion) and right coronary artery (4 lesions), double-vessel disease was identified in 67% (single/double/triple = 2/4/0), and 41% of the subjects (n = 16/39) had the metabolic syndrome based on visceral fat accumulation. Four subjects with CAD (n = 4/6) underwent successful revascularization with percutaneous coronary intervention procedures, but not coronary artery bypass graft surgery, and the other two subjects had taken medications only.

**Table 1:** Baseline characteristics of the subjects (males, n = 39)

	mean ± SEM (%)	range
Age (years)	52 ± 1	(35-74)
Body mass index (kg/m^2^)	27.8 ± 0.6	(20.2-40.3)
Neck circumference (cm)	40 ± 0.4	(36-46)
Waist circumference (cm)	95 ± 1	(80-114)
Hip circumference (cm)	100 ± 1	(90-116)
Total fat area (cm^2^)	330 ± 35	(86-1313)
Visceral fat area (cm^2^)	163 ± 31	(22-1117)
Subcutaneous fat area (cm^2^)	167 ± 9	(64-312)
Smoking (none-/ex-/current-smoker)	13/14/12	
Systolic blood pressure (mmHg)	131 ± 2	(100-160)
Diastolic blood pressure (mmHg)	82 ± 2	(58-108)
Fasting blood glucose (mg/dL)	80 ± 3	(61-124)
Fasting immunoreactive insulin (μIU/mL)	11 ± 1	(3-26)
Triglyceride (mg/dL)	158 ± 12	(52-375)
High-density lipoprotein-cholesterol (mg/dL)	49 ± 2	(33-100)
Low-density lipoprotein-cholesterol (mg/dL)	133 ± 3	(97-171)
Diabetes mellitus/Hypertension/Dyslipidemia	3/28/28	
Metabolic syndrome	16 (41)	
Epworth Sleepiness Scale	13 ± 1	(2-23)
Cardiorespiratory monitoring findings		
Apnea-hypopnea index (AHI) (events/hour)	39 ± 4	(8.2-116.6)
Baseline SpO_2 _(%)	98 ± 0.1	(97-100)
Lowest SpO_2 _(%)	72 ± 2	(33-91)
4% oxygen desaturation index (events/hour)	27 ± 3	(1.4-91.1)
% < 90% time	7 ± 2	(0-51.4)
Apnea-hypopnea index (AHI) ≥ 5 to < 15 (mild)	7	
≥ 15 to < 30 (moderate)	11	
≥ 30 (severe)	21	
Computed tomography scan findings		
< 50% stenosis of coronary arteries	33 (75)	
≥ 50% stenosis of coronary arteries	6 (15)	
Serum high-sensitive C-reactive protein (hsCRP) (ng/dL)	2131 ± 830	(96-29100)
Serum thiobarbituric acid reactive substance (TBARS), nmol/mL	5.5 ± 0.2	(2.7-8.0)
Serum adiponectin (μg/mL)	5.6 ± 0.5	(2.1-11.8)

### Relationship between CAD and various parameters

Logistic regression analysis was performed to evaluate the relation between CAD and each parameter listed in Table 2. Simple logistic regression analysis showed a significant relationship between age-adjusted CAD and metabolic syndrome as well as TBARS (*p *< 0.05), but not adiponectin.

**Table 2:** Results of simple logistic analysis for coronary artery disease (CAD)

	Model 1	Model 2
Parameter	p value	p value
Age	0.079	-
Body mass index	0.538	0.329
Neck circumference	0.224	0.164
Waist circumference	0.196	0.134
Hip circumference	0.477	0.365
Log-Total fat area*	0.871	0.414
Log-Visceral fat area*	0.555	0.239
Log-Subcutaneous fat area*	0.671	0.905
Smoking (Ex+current-smoker)	1.000	0.965
Systolic blood pressure	0.631	0.868
Diastolic blood pressure	0.870	0.457
Fasting blood glucose	0.052	0.275
Fasting immunoreactive insulin	0.824	0.671
Log-Triglyceride*	0.702	0.734
High-density lipoprotein-cholesterol	0.354	0.298
Low-density lipoprotein-cholesterol	0.466	0.445
AHI	0.850	0.882
Metabolic syndrome	**0.033**	**0.047**
Log-hsCRP*	0.267	0.107
Log-TBARS*	**0.014**	**0.039**
Log-adiponectin*	0.503	0.928

Significant level was set at *p *value < 0.05 (bold type). Abbreviation was shown as in Table 1 *log-transformed. Model 1: non-adjusted simple logistic analysis, Model 2: age-adjusted simple logistic analysis

### Comparison of parameters in subjects without and with the metabolic syndrome

**Figure 1:**
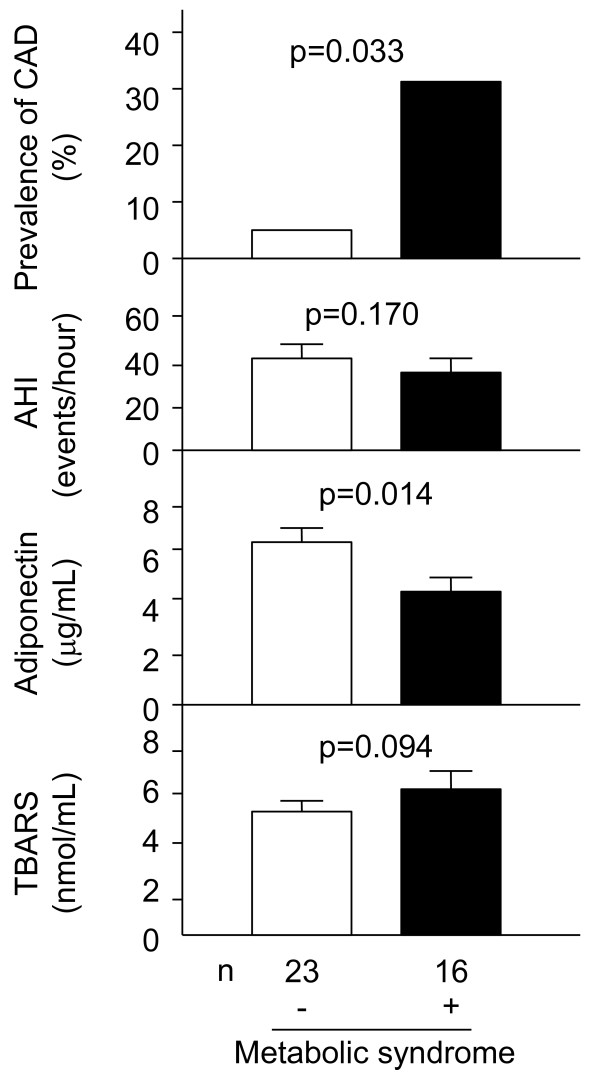
**Comparisons of the prevalence of CAD, AHI and circulating levels of adiponectin and TBARS between SDB/OSA subjects without and with the metabolic sydnrome**. CAD, coronary artery disease; AHI, apnea-hypopnea index; SDB, sleep-disordered breathing; OSA, obstructive sleep apnea; TBARS, thiobarbituric acid reactive substance. All values were expressed as mean ± SEM, and compared by the *χ*^2 ^and Mann- Whitney *U*-test in experiments of two groups. In all cases, *p *values < 0.05 was considered statistically significant.

Subjects with SDB/OSA were divided into two groups based on the metabolic syndrome [21]. The metabolic syndrome group had significantly higher prevalence of CAD and lower levels of serum adiponectin, compared with groups without the metabolic syndrome (Figure 1; 31.3 versus 4.3%, *p *= 0.033, 4.5 ± 0.6 versus 6.4 ± 0.6 μg/mL, *p *= 0.014). There was no significant difference of AHI and TBARS in two groups (Figure 1; 32.6 ± 5.8 versus 42.7 ± 5.7 events/hour, *p *= 0.170; 5.2 ± 0.3 versus 6.0 ± 0.4 nmol/mL, *p *= 0.094).

## Discussion

The two major findings of the present study of SDB/OSA subjects with a history of snoring and/or witnessed apneas were: 1) Identification of greater than 50% intracoronary narrowing in 15% (6/39) of total, and in 4.3% (1/23) of subjects without the metabolic syndrome and in 31.3% (5/16) of those with the metabolic syndrome. Differences in the above rates were significant (*p *= 0.033). 2) The metabolic syndrome as well as systemic oxidative stress, i.e. TBARS, correlated with intracoronary stenosis detected by MSCT. However, multiple logistic regression analysis (adopted factors; age, metabolic syndrome, TBARS), did not identify both metabolic syndrome and TBARS as significant and independent determinants of CAD (*p *= 0.366, *p *= 0.062, respectively). The study included a limited number of patients and further replication studies of larger sample need to be designed in the future. Our results may provide a rationale to conduct such studies.

Although SDB/OSA is associated with cardiovascular diseases [1-3], there is little information on SDB/OSA subjects with CAD remain to be understood. The present study showed that metabolic syndrome predicts intracoronary stenotic lesions in SDB/OSA subjects. A cluster of multiple risk factors based on visceral fat accumulation accelerates atherosclerogenesis, i.e. metabolic syndrome [23]. The co-existence of metabolic syndrome and SDB/OSA based on visceral fat accumulation, i.e. "Syndrome Z", may represent two sides of the same coin, given the common pathophysiological processes evident in both conditions, such as insulin resistance and atherosclerosis [7,15]. Although adiponectin *per se *did not correlate with CAD in SDB/OSA subjects, serum adiponectin levels were lower in subjects with the metabolic syndrome than those without the metabolic syndrome. Metabolic syndrome was related with serum levels of adiponectin (*p *= 0.029), but not with hsCRP and TBARS (*p *= 0.850, *p *= 0.102, respectively). The results suggest that the metabolic syndrome and hypoadiponectinemia may play a role in the development of CAD in subjects with SDB/OSA. The metabolic syndrome and hypoadiponectinemia might be a target for prevention of potential CAD in SDB/OSA.

Nasal continuous positive airway pressure (CPAP) is the "gold standard" treatment of OSA. A recent double-blind, placebo-controlled, randomized, and crossover trial reported that 3 months of nasal CPAP therapy improved control of elevated blood pressure and a reduction in metabolic abnormalities including abdominal obesity [24]. Nasal CPAP also reduces the risk of fatal and non-fatal cardiovascular outcomes [25]. However, whether visceral fat reduction prevents cardiovascular events in SDB/OSA subjects remains unclear. Longer observational and interventional trials should be conducted to assess the effect of visceral fat reduction on the incidence of cardiovascular events in SDB/OSA subjects.

## Conclusion

We described that metabolic syndrome was correlated with intracoronary stenosis detected by MSCT in Japanese SDB/OSA subjects, suggesting that any program designed to prevent cardiovascular diseases in SDB/OSA subjects should include continuous evaluation of metabolic syndrome.

## Study limitations

The present study has several limitations. First, all patients in this study were Japanese males and any differences from other ethnicities are unknown. Second, there is bias in single center trials. Finally, based upon 80% power to detect statistically significant correlations between CAD and the various parameters (*p *= 0.05; two-sided, r = 0.5, α = 0.05, 1-β = 0.8), a sample size of at least 38 patients was required to demonstrate. The number of patients may be still small, and further investigations might be required to confirm the present results.

## Abbreviations

AHI: Apnea-hypopnea index; CAD: Coronary artery disease; CPAP: Continuous positive airway pressure; CT: Computed tomography; CV: Coefficients of variation; ESS: Epworth Sleepiness Scale; hs-CRP: high-sensitivity C-reactive protein; MSCT: multislice computed tomography; OSA: obstructive sleep apnea; SDB: sleep-disordered breathing; TBARS: thiobarbituric acid-reacting substance.

## Competing interests

Ken Kishida and Tohru Funahashi are members of the "Department of Metabolism and Atherosclerosis", a sponsored course endowed by Kowa Co. Ltd. and a company researcher is dispatched to the course. All other authors declare no competing interests.

## Authors' contributions

TN-M and KK researched and analyzed data. KK also participated in the concept and design of the study, interpretation of data and reviewed/edited the manuscript. YN, MN, CN, YN and KY collected the data. RY recruited the patients. TF and IS contributed to discussion and wrote the manuscript. All authors read and approved the final version of the manuscript.
